# Supplementary material for: A comprehensive atlas of fetal splicing patterns in the brain of adult myotonic dystrophy type 1 patients
Source: NAR Genom Bioinform. 2022 Mar 8;4(1):lqac016. doi: 10.1093/nargab/lqac016 (PMC8903011; doi:10.1093/nargab/lqac016)

Supplemental Figure S1. Exon inclusion does not differ significantly between subregions of the frontal cortex during human brain development.
Differences in Ψ are shown for the selection of splice events in Figure 2. The boxplots are based on samples from frontal cortex subregions of DM1-unaffected prenatal and postnatal donors. A rank-sum test showed no significant difference between any subregions within the prenatal or postnatal group ($p > 0.05$). P-values were FDR-corrected with the Benjamini-Hochberg procedure.

Supplemental Figure S2. Sashimi plots of selected examples for three splice events from four of the samples analyzed in this study. Coverage and junction spanning reads are shown for differentially spliced exons in *ADD1*, *DMD* and *SORBS1* in a DM1 and control sample from the Otero et al. (2021) dataset (SRR12582121, red; SRR12582150, blue) and samples obtained 84 and 7215 days post-conception from the BrainSpan dataset (SRR3583535, prenatal green; SRR3583673, adult, brown) (23, 78). The images were generated using Integrative Genomics Viewer (85).

Supplemental Figure S3. Developmentally-regulated splice events show on average larger differences in Ψ between DM1 patients and unaffected controls compared to all DM1-related events. The absolute Ψ difference is shown for 130 splice events that were found to be significantly different when comparing frontal cortex samples from DM1 patients and unaffected controls ($|\Delta\Psi| > 0.2$, $p < 0.01$ by rank-sum test). Splice events with a significant Ψ difference between prenatal and postnatal samples of the healthy, developing brain are colored in blue and marked by the letter “x”.

Supplemental Figure S4. Splice events show negligible difference in exon inclusion between the sexes. Representation as in Figure 2 but separated by sex of the sample donor.

Supplemental Figure S5. Exon inclusion varies between CNS regions in the healthy, adult brain. Differences in Ψ are shown for a selection of splice events with the largest decrease (A) or increase (B) in Ψ in DM1 patients compared to unaffected adults. The boxplots are based on samples from various CNS regions of unaffected, adult donors. No significant Ψ difference was found between control frontal cortex samples from Otero et al. (2021) and GTEx. The following regions are shown: FrnC=Frontal cortex (Otero et al., 2021: n=8; GTEx: n=54), Ancc=Anterior cingulate cortex (n=15), Hppc=Hippocampus (n=15), Hypt=Hypothalamus (n=14), Spnc=Spinal cord (n=14), Ptmn=Putamen (n=15), Ncla=Nucleus accumbens (n=15), CrbH=Cerebellar Hemisphere (n=15), Cadt=Caudate (n=15).

Supplemental Figure S6. RNA expression of *CELF* family genes throughout human brain development and between DM1 patients and unaffected adults. Representation as in Figure 3.

Supplemental Figure S7. RNA expression of *MBNL* family genes throughout human brain development and between DM1 patients and unaffected adults. Representation as in Figure 3.

Supplemental Figure S8. RNA expression of DM1-relevant splicing factors differs between sexes throughout human brain development but not between DM1 patients and unaffected adults. Representation as in Figure 3 but separated by sex of the sample donor.

Supplemental Figure S9. Correlation coefficients for correlation between high-confidence splice events and splicing factors expression. Representation as in Figure 4, supplemented with the value of the Spearman’s rank correlation coefficients.

Supplemental Figure S10. Scatter plots of *ADD1* and *DMD* exon inclusion versus RNA expression of *CELF1*, *MBNL1* and *MBNL2*. The Ψs of two exemplary splice events (i.e. *ADD1* and *DMD*) are plotted against the RNA expression of *CELF1*, *MBNL1* and *MBNL2.* The scatter plots are based on frontal cortex samples from the healthy, developing brain (A) and from DM1 patients and unaffected controls

(B). The saturation of the colored squares illustrates the strength of the correlation as in the heatmap in Figure 4. Asterisks indicate a significant correlation (FDR-corrected $p < 0.05$).

Supplemental Figure S11. Mixed data from prenatal and postnatal sample donors is necessary to reveal all correlational patterns between the splice events. Correlation between the Ψs of high-confidence exon skipping events and RNA expression levels of *CELF1, MBNL1* and *MBNL2.* Correlations are displayed for frontal cortex samples from the healthy, developing brain before (left) and after (right) birth, along with their combination identical to the left panel in Figure 4 (left). Representation as in Figure 4.

Supplemental Figure S12. Correlation between exon inclusion of non-developmental DM1-relevant splice events and RNA expression of *CELF1*, *MBNL1* and *MBNL2*. Correlations were computed between Ψ of exon skipping events and RNA expression levels of *CELF1, MBNL1* and *MBNL2* within the same samples from the frontal cortex of DM1 patients and unaffected adults. Events on the left show an increased inclusion in DM1 patients whereas events on the right show a decreased inclusion. Exon inclusion for this set of events is significantly different between DM1 patients and unaffected controls ($|\Delta\Psi| > 0.2$, $p < 0.01$ by rank-sum test) but not between prenatal and postnatal samples in the developmental dataset. The color scale on the right reflects the value of the Spearman's rank correlation coefficient. Asterisks indicate a significant correlation (per study FDR-corrected $p < 0.05$).

Supplemental Figure S13: Mixed data from DM1 patients and unaffected adults is necessary to reveal weak correlational patterns between the splice events. Correlation between the Ψs of high-confidence exon skipping events and RNA expression levels of *CELF1, MBNL1* and *MBNL2.* Correlations are displayed separately for frontal cortex samples from DM1 patients (middle) and unaffected adults (right), along with their combination identical to the left panel in Figure 4 (left). Representation as in Figure 4.

Supp. Figure S1

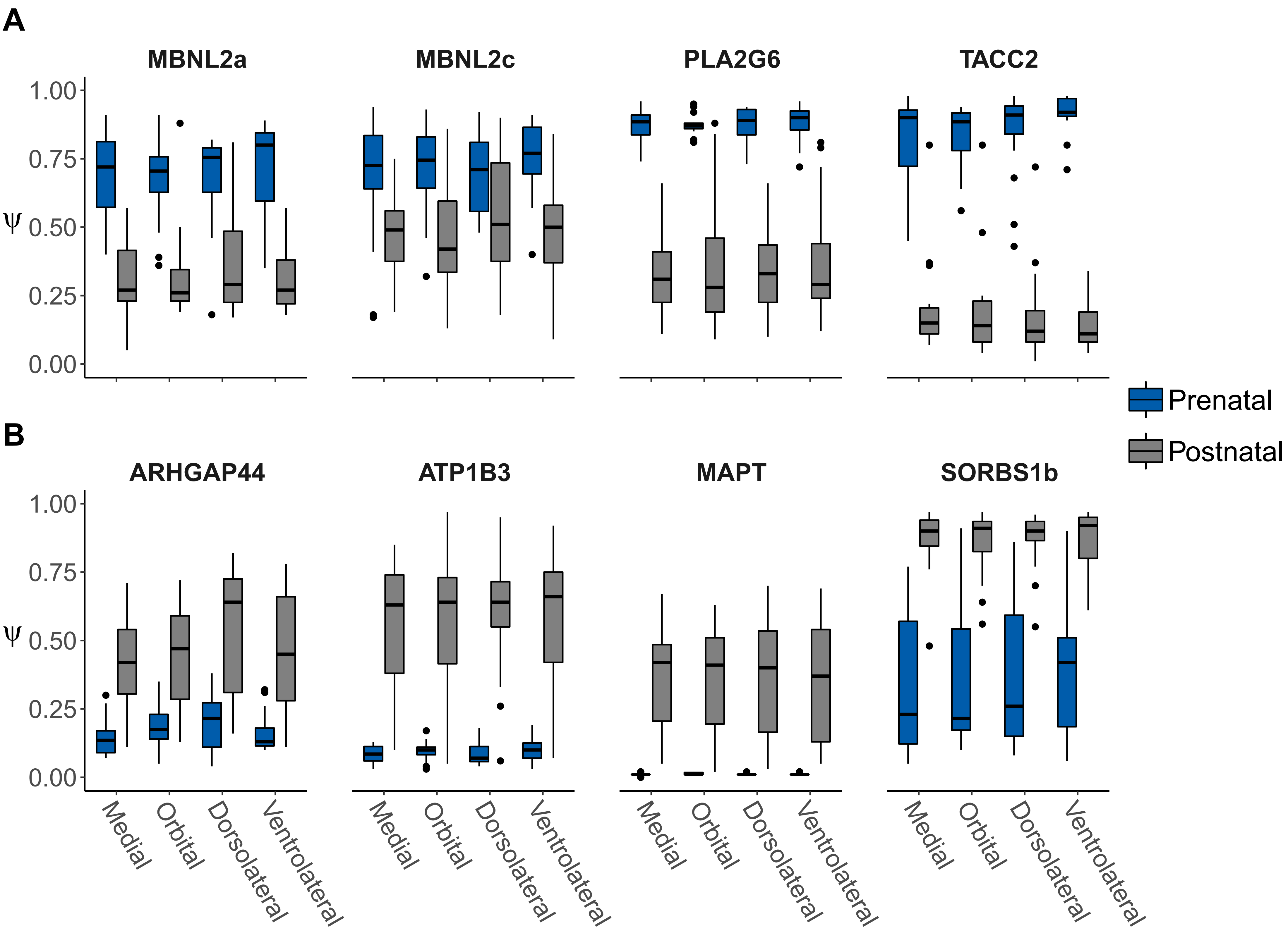

Supp. Figure S2

ADD1

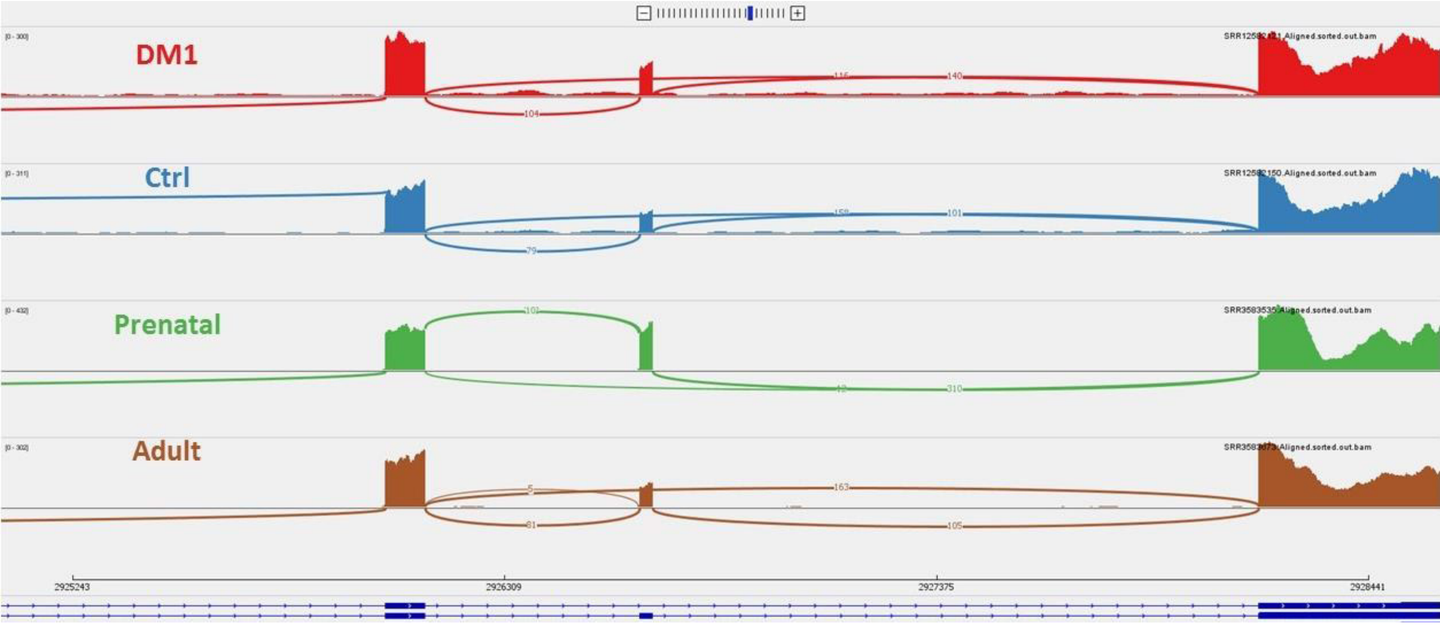

DMD

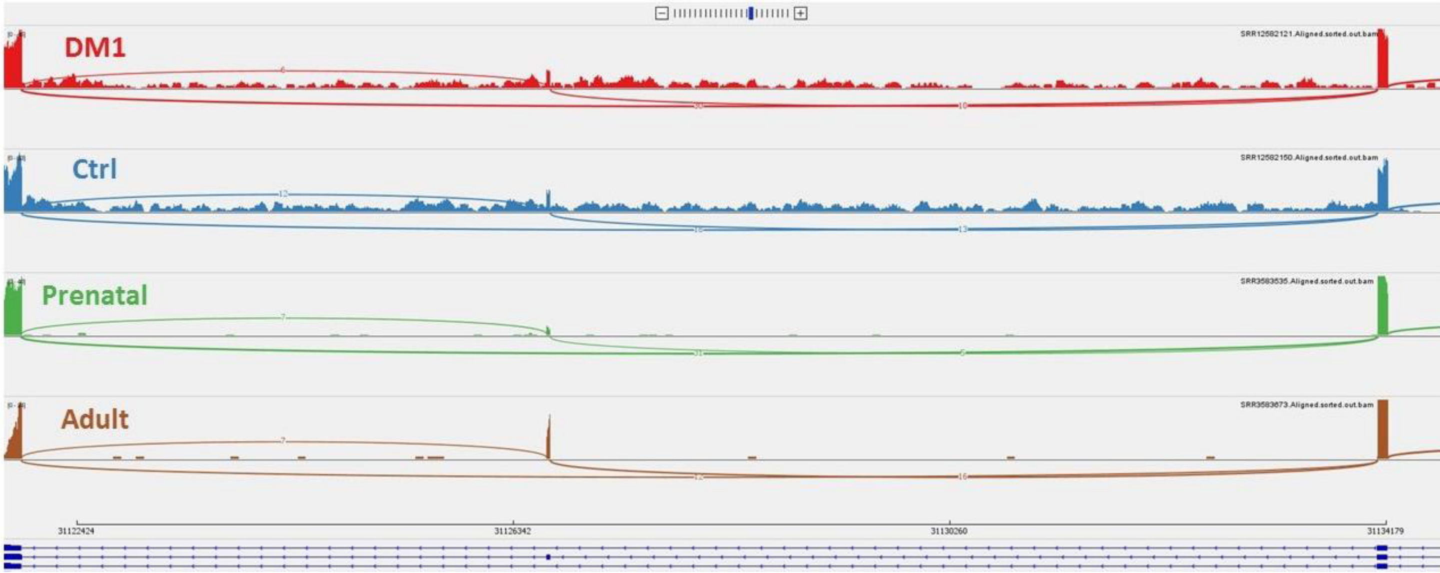

SORBS1

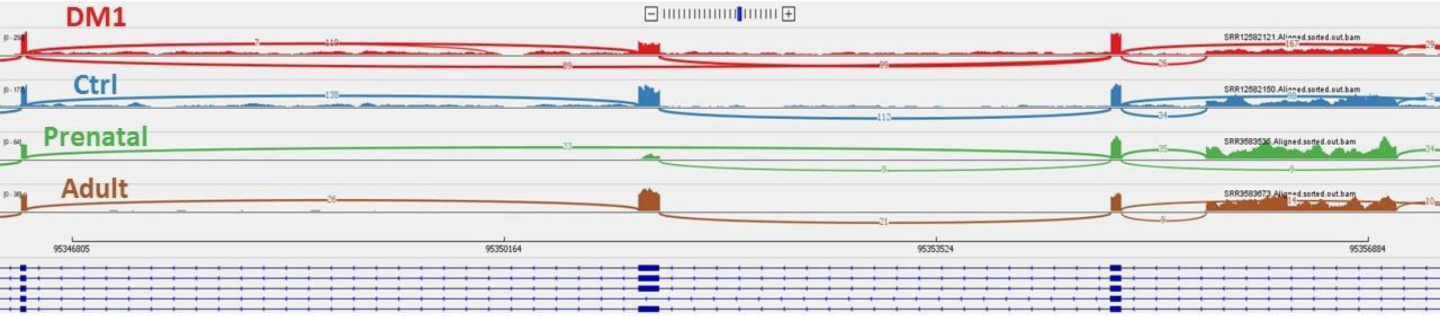

Supp. Figure S3

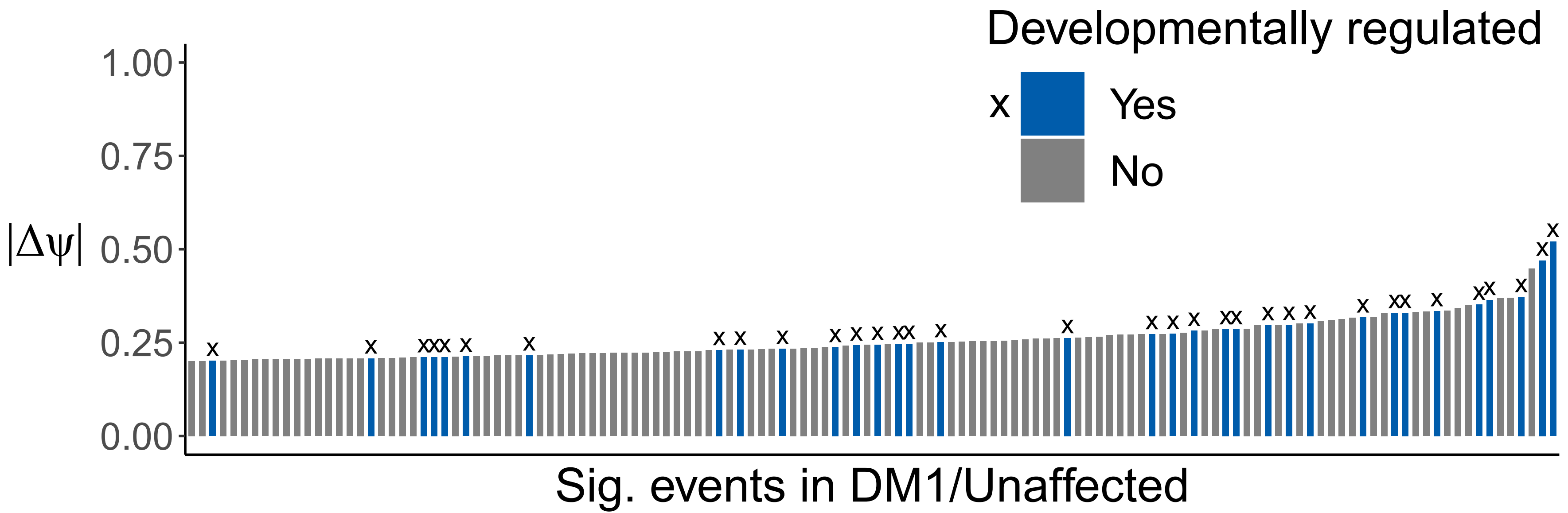

Supp. Figure S4

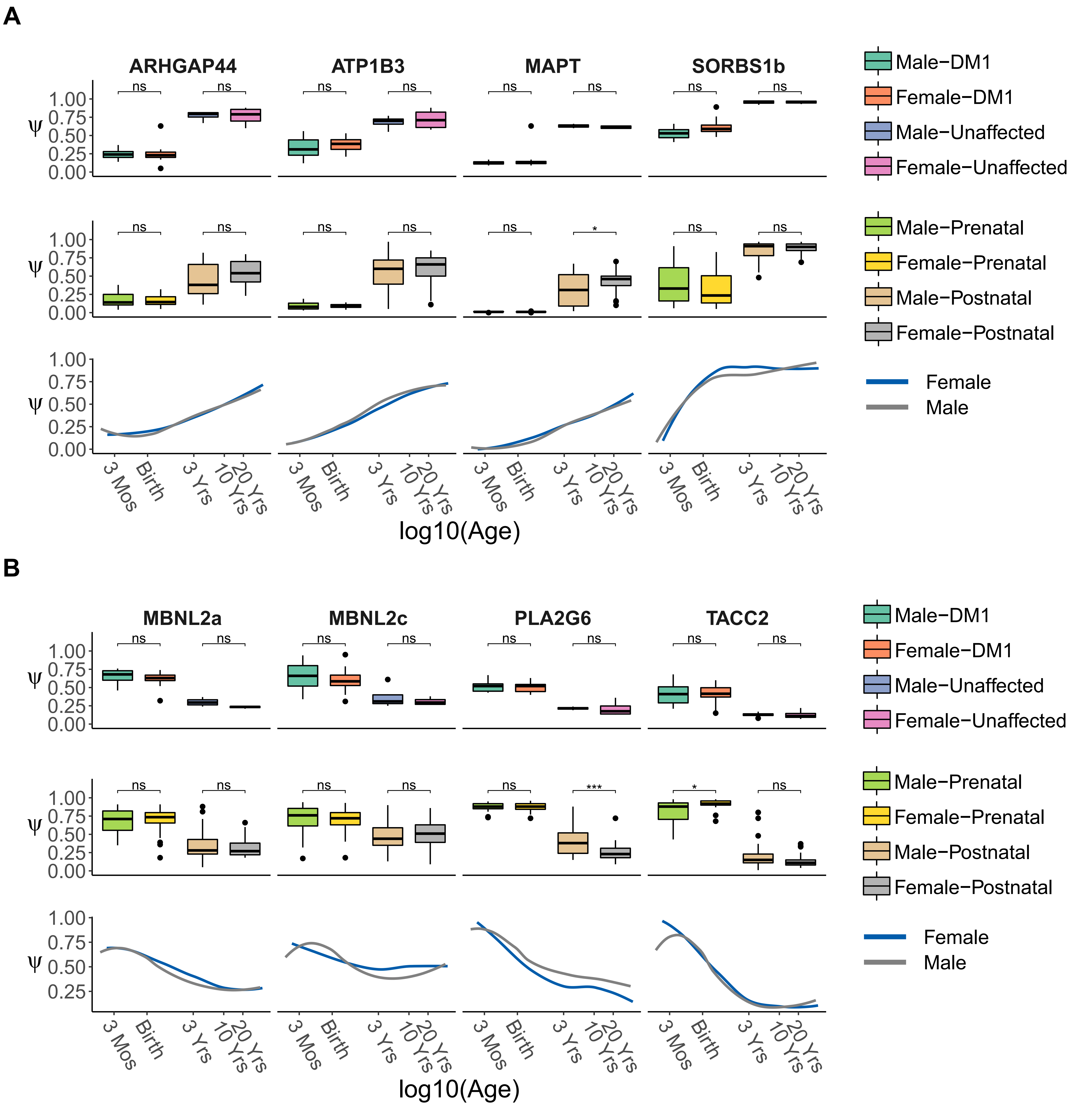

Supp. Figure S5

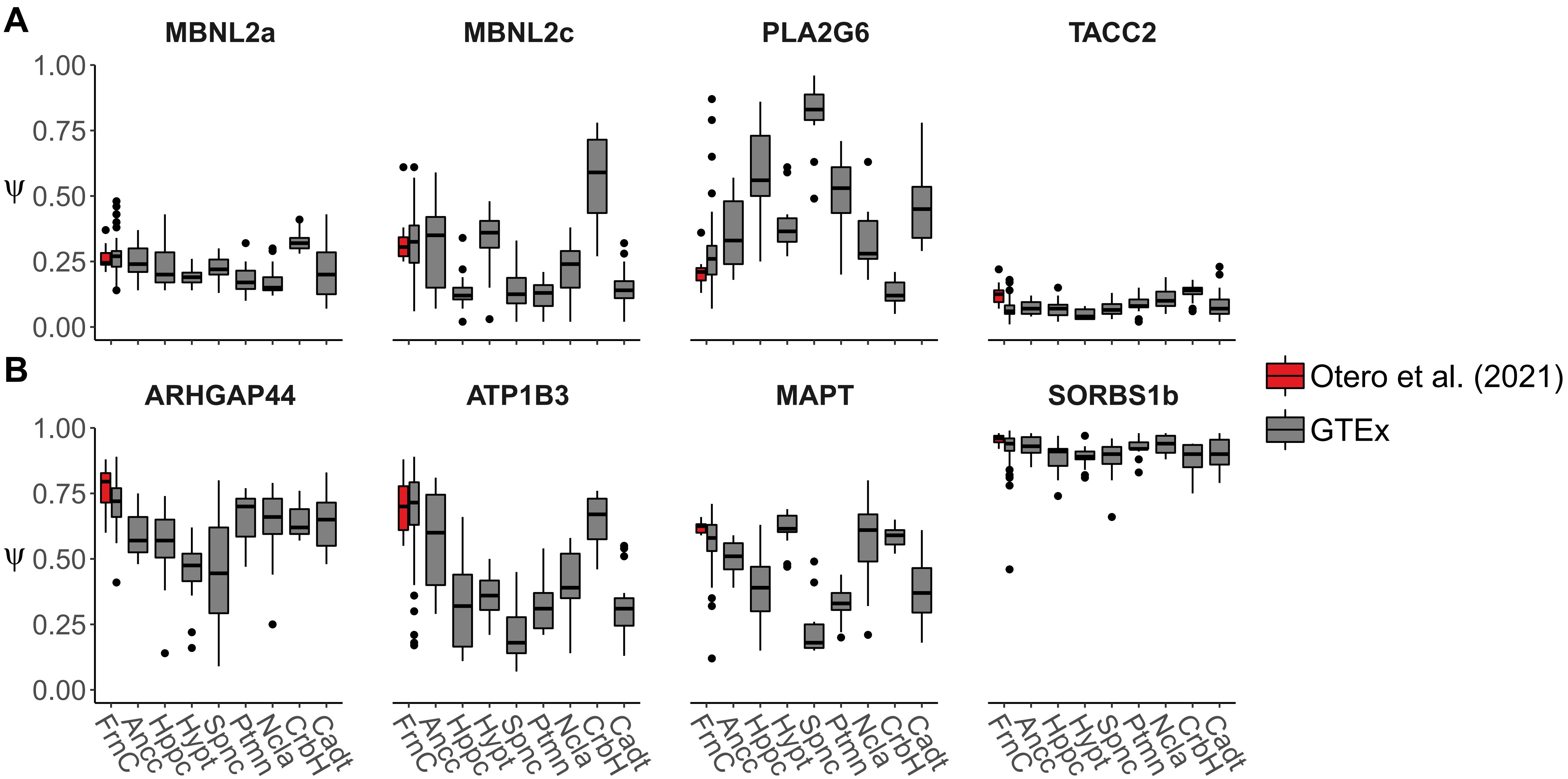

Supp. Figure S6

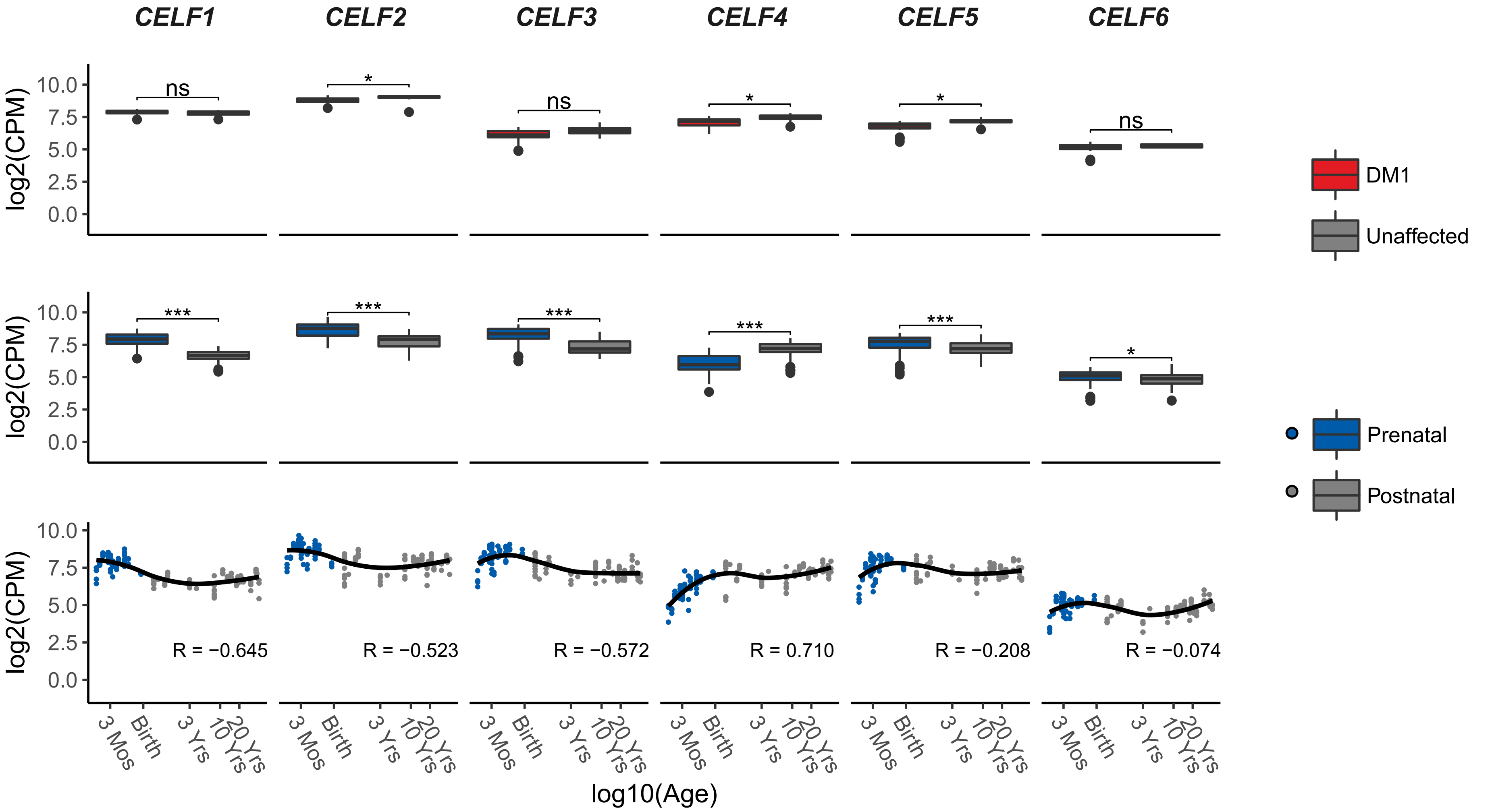

Supp. Figure S7

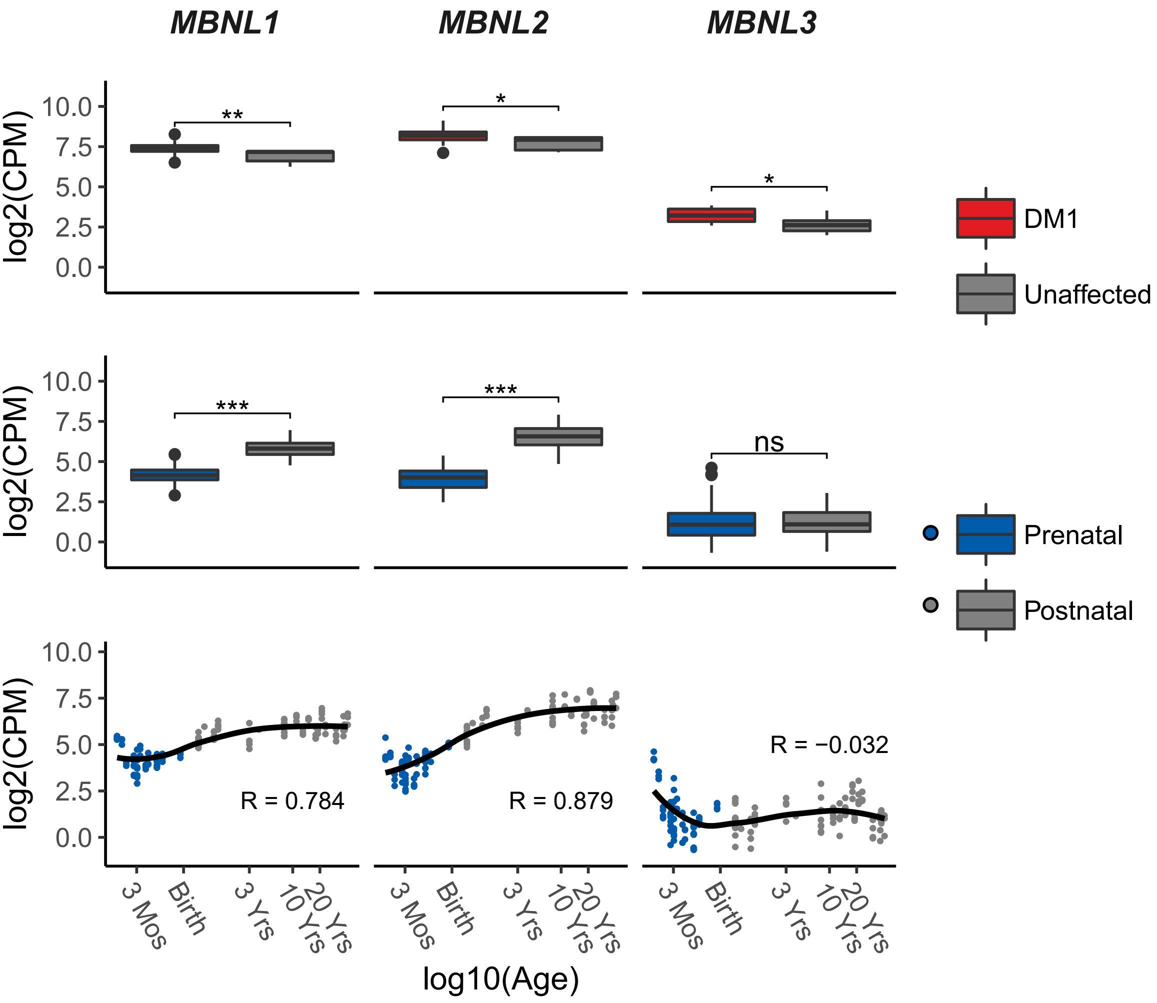

Supp. Figure S8

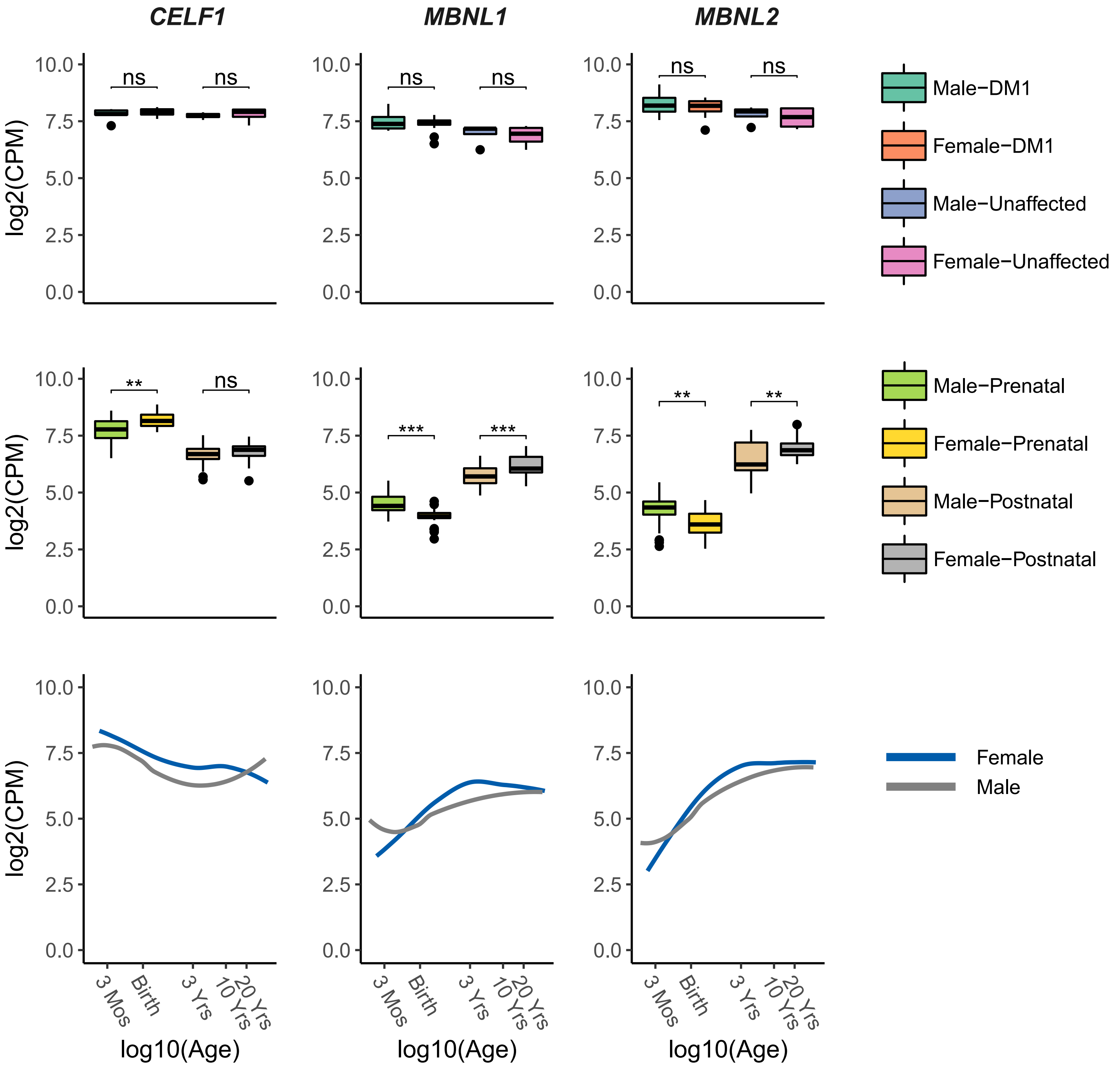

Supp. Figure S9

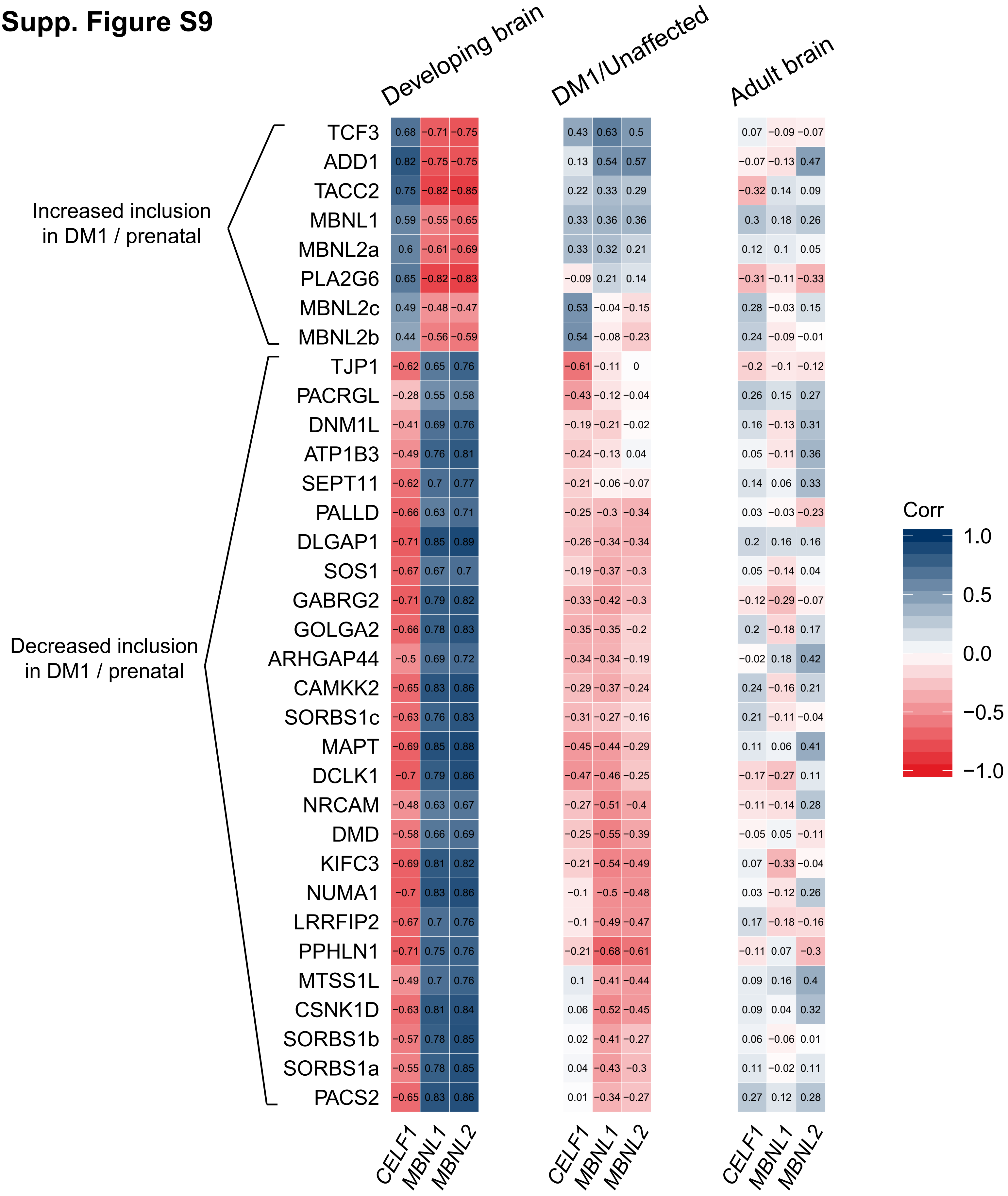

Supp. Figure S10

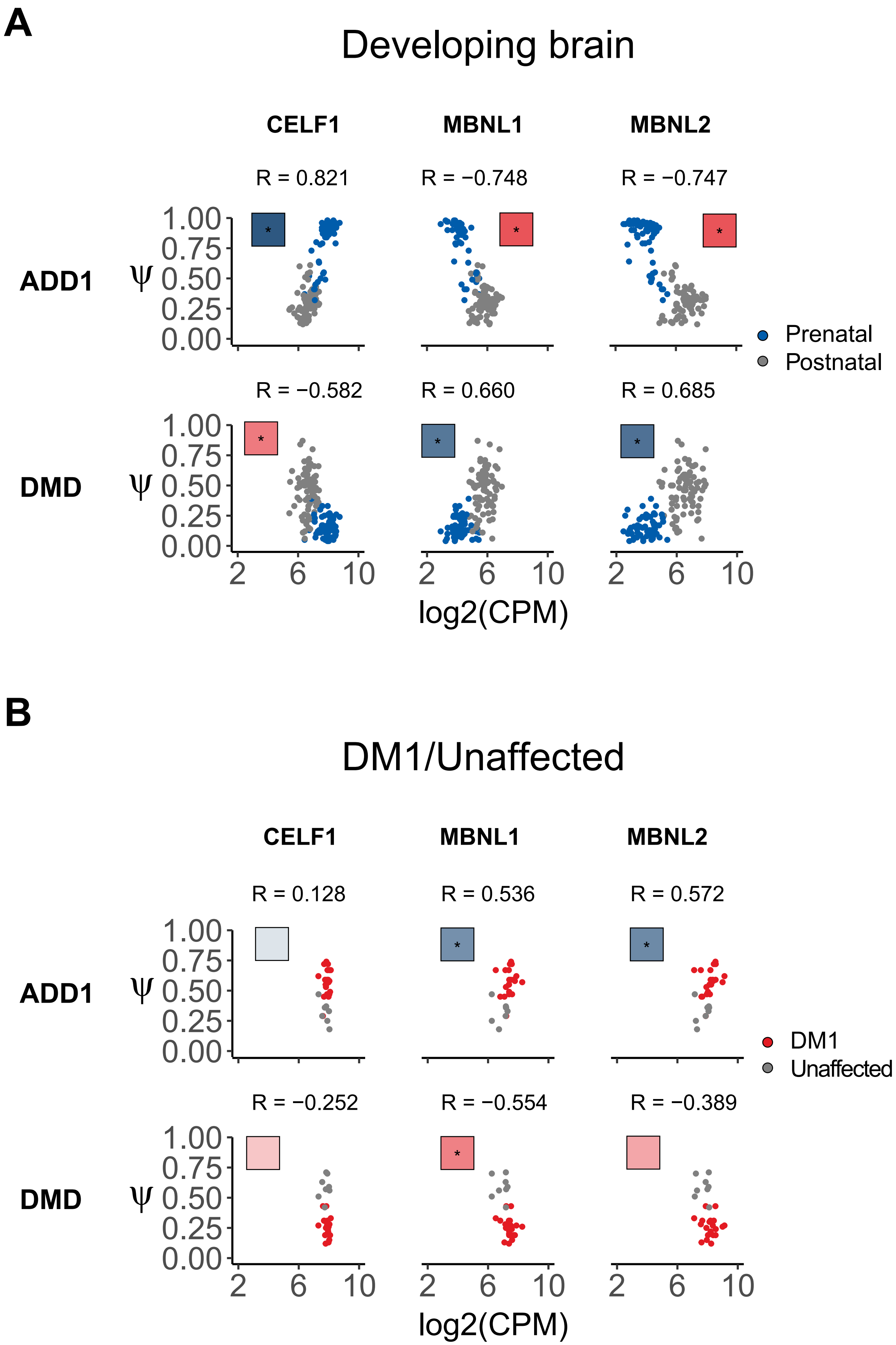

Supp. Figure S11

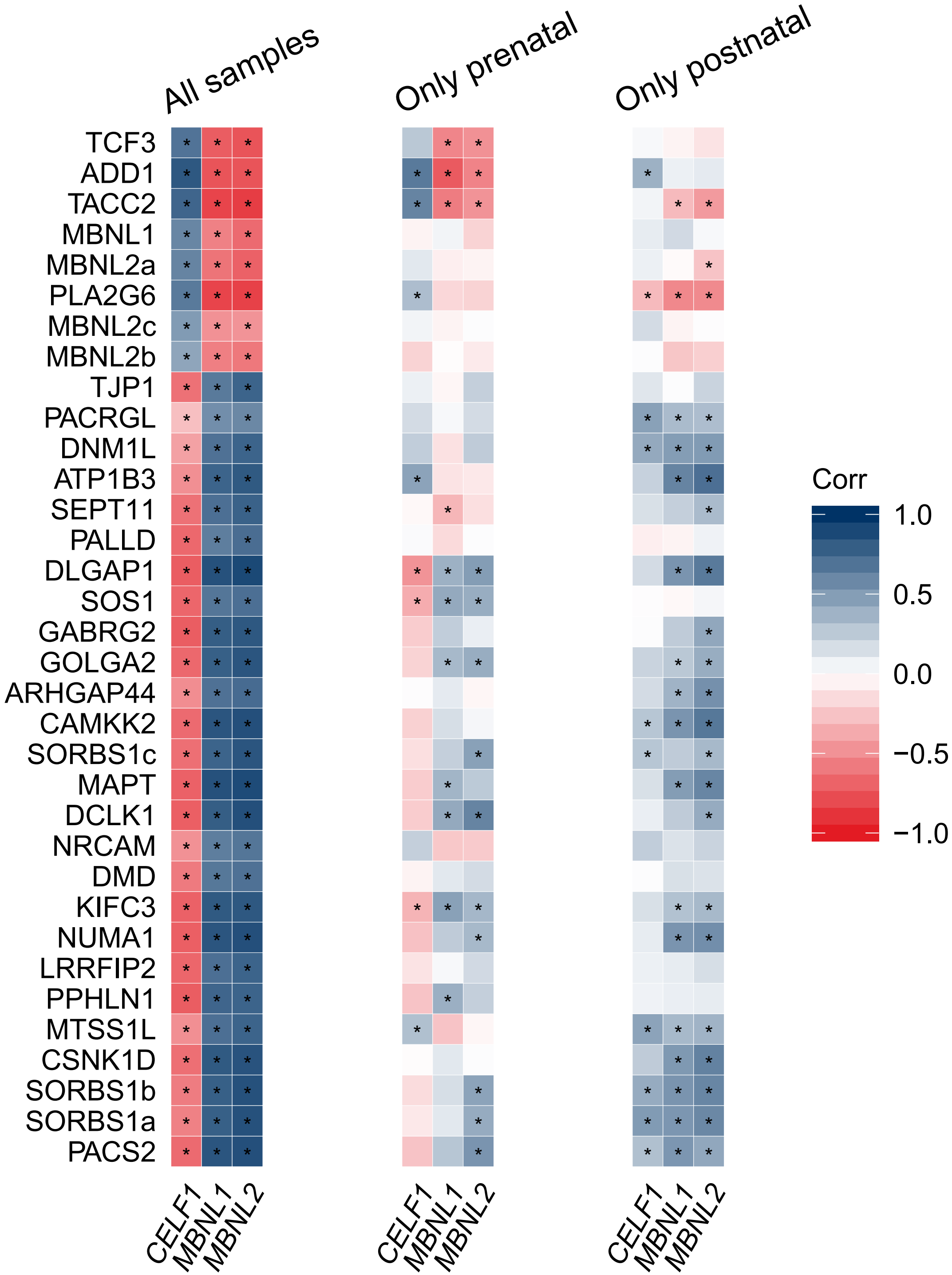

Supp. Figure S12

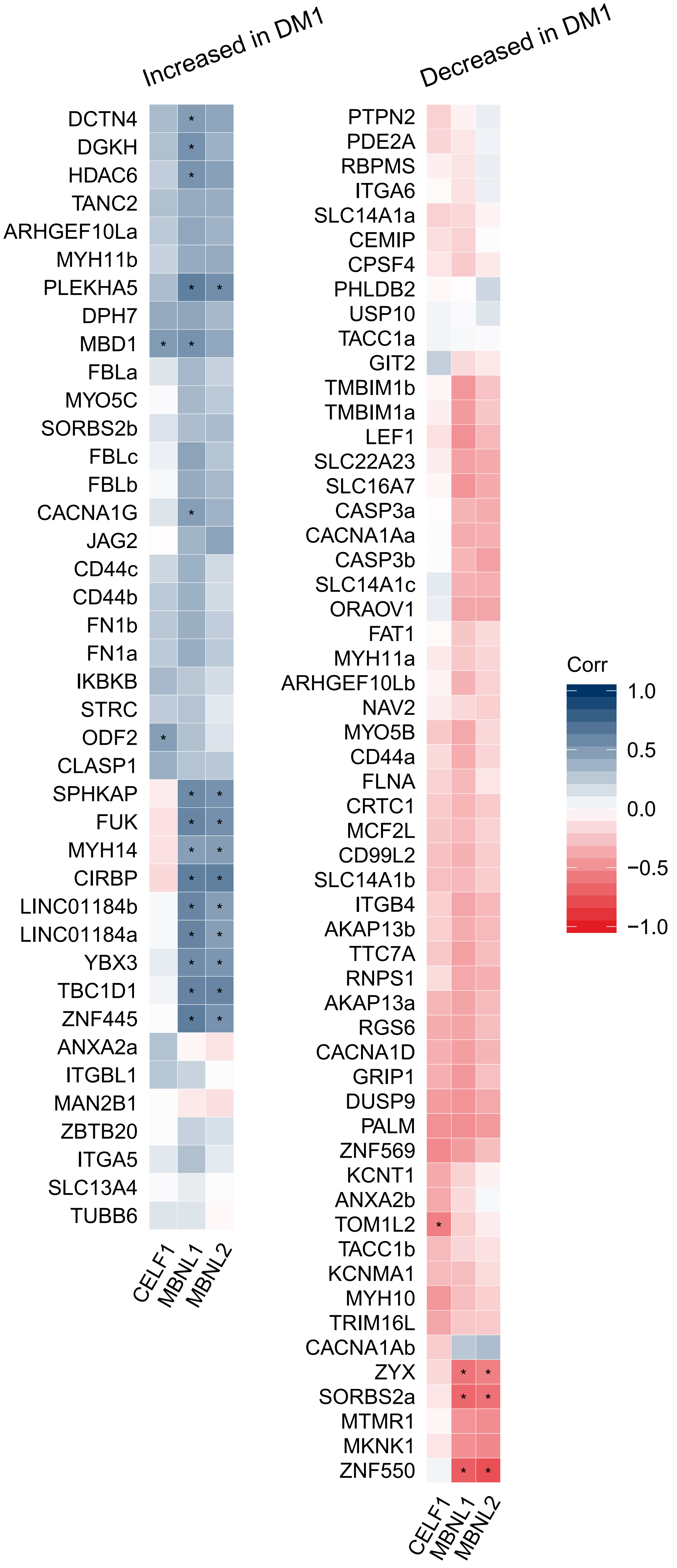

Supp. Figure S13

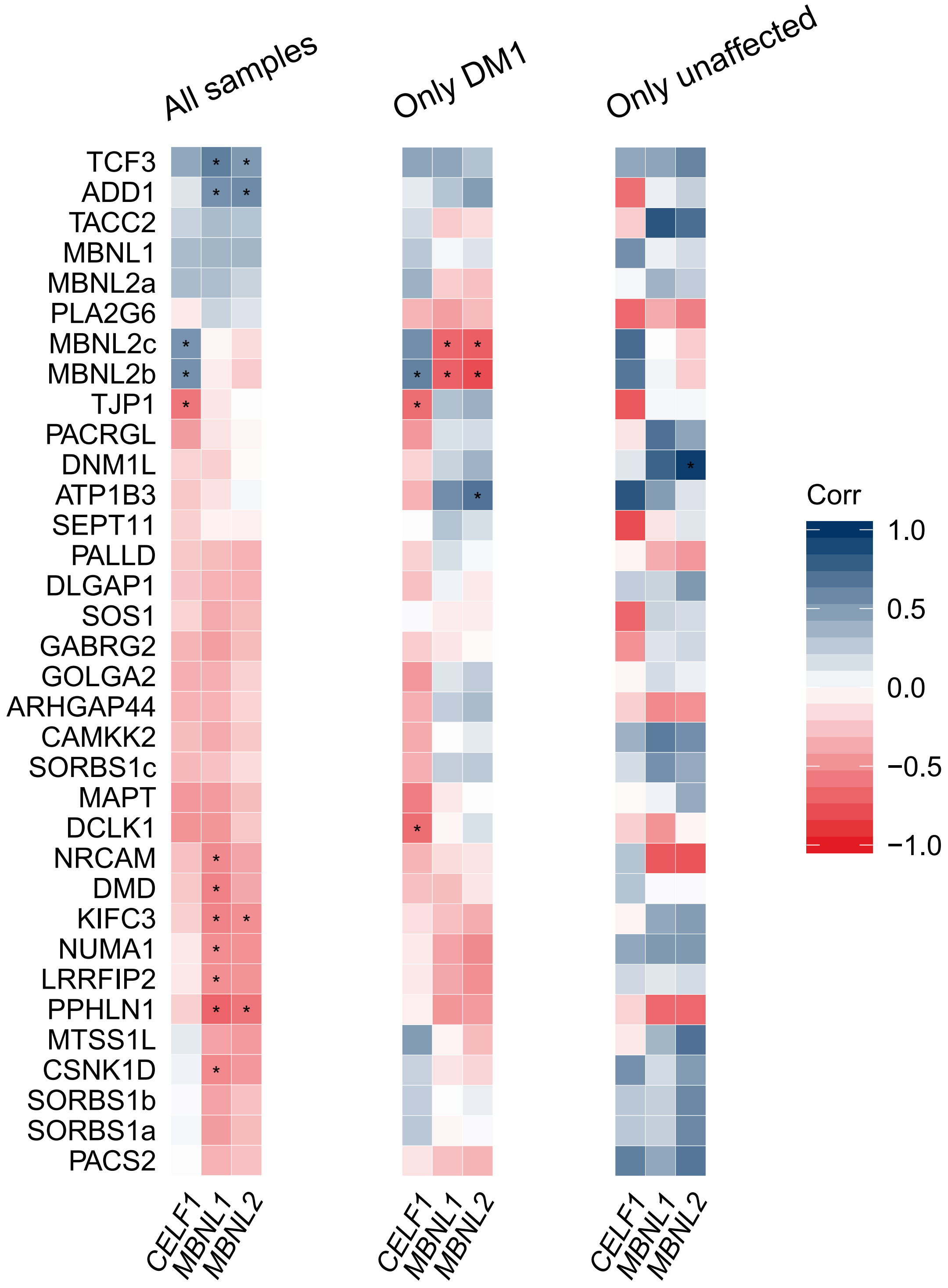

Supplement: lqac016_Supplemental_Files [file lqac016_supplemental_files.zip › SupplFigures_complete.pdf]
